# Fungal-mediated lung allergic airway disease: The critical role of macrophages and dendritic cells

**DOI:** 10.1371/journal.ppat.1010608

**Published:** 2022-07-14

**Authors:** Julio Furlong-Silva, Peter Charles Cook

**Affiliations:** Medical Research Council Centre for Medical Mycology, University of Exeter, Exeter, United Kingdom; Universitat Zurich, SWITZERLAND

## Abstract

Fungi are abundant in the environment, causing our lungs to be constantly exposed to a diverse range of species. While the majority of these are cleared effectively in healthy individuals, constant exposure to spores (especially *Aspergillus* spp.) can lead to the development of allergic inflammation that underpins and worsen diseases such as asthma. Despite this, the precise mechanisms that underpin the development of fungal allergic disease are poorly understood. Innate immune cells, such as macrophages (MΦs) and dendritic cells (DCs), have been shown to be critical for mediating allergic inflammation to a range of different allergens. This review will focus on the crucial role of MΦ and DCs in mediating antifungal immunity, evaluating how these immune cells mediate allergic inflammation within the context of the lung environment. Ultimately, we aim to highlight important future research questions that will lead to novel therapeutic strategies for fungal allergic diseases.

## Introduction

Fungi are abundant in our environment, which leads to a large amount of fungal material being breathed into lungs on a daily basis [1]. Many individuals clear these fungi with no apparent sign of disease, but can trigger the development of allergic inflammatory diseases [2–4] such as severe fungal sensitised asthma [5–7] estimated to impact 10 million people worldwide [8]. Despite this, the underlying mechanism(s) that cause fungi to mediate these chronic diseases are poorly understood.

A variety of cell types in the lung have been shown to trigger responses to environmental allergens that causes allergic inflammation. In particular, myeloid innate immune cells such as macrophages (MΦ) and dendritic cells (DCs) have been shown to be essential [9–11]. However, these cells are also crucial for the clearance of fungal spores, to prevent tissue penetration leading to invasive disease [12]. The underlying events that cause MΦ and DCs to switch from orchestrating spore clearance (maintaining a “healthy environment”), to mediating diseases such as severe asthma are poorly defined.

Several excellent reviews have previously highlighted the clinical burden of fungal asthma and the general immune mechanism(s) that underpin the development of allergic inflammation to fungi [12–15]. Therefore, the aim of this review is to assess our current understanding of the unique role MΦs and DCs play in directing and maintaining fungal allergic inflammation. We will reflect how this improves our appreciation of fungal allergic inflammation and highlight the challenges that remain.

### The global health impact of fungal driven asthma

There are approximately 300 million people with asthma worldwide, and this is expected to rise to 400 million by 2025, placing a huge burden on global health [16,17]. Fungi such as *Aspergillus* spp. can trigger a spectrum of allergic airway inflammatory diseases, ranging from asthma, allergic fungal rhinosinusitis (AFRS), allergic bronchopulmonary aspergillosis (ABPA), and severe asthma with fungal sensitisation (SAFS) (Table 1) [3,18]. It is estimated that up to 10 million people globally suffer from severe asthma, as a direct result of hypersensitivity towards *Aspergillus fumigatus (Af*) [8]. Typically 1000s of spores are inhaled daily [19–21], and if spore clearance fails (typically in immunocompromised situations), fungi can grow and invade the lung tissue, causing invasive Aspergillosis [22,23]. Therefore, a delicate balance of appropriate responses to clear fungal spores, while avoiding hypersensitivity, is required to maintain a healthy lung barrier.

**Table 1:** List of abbreviations and acronyms.

Abbreviation	Name
ABPA	Allergic bronchopulmonary aspergillosis
*Af*	*Aspergillus fumigatus*
AFRS	Allergic fungal rhinosinusitis
Alp 1	Alkaline protease 1
AlvMΦ	Alveolar macrophage
Aspf13	*Aspergillus* protease allergen
BATF(number)	Basic leucine zipper ATF-like transcription factor
Ca^2+^	Calcium ion
CCR(number)	C-C motif chemokine receptor
CD(number)	Cluster of differentiation (number)
cDC	Conventional dendritic cells
CLC	Charcot–Leyden crystals
CLEC (number)	C-type lectin domain containing (number)
CLR	C-type lectin receptors
CXCL(number)	Chemokine (C-X-C motif) ligand
DAMP	Damage-associated molecular pattern
DC	Dendritic cell
DNGR (number)	CLEC9A
FAO	Fatty acid oxidation
FCP	Fibrinogen cleavage products
FcεR(number)	Fc epsilon receptor (number)
FleA	*A*. *fumigatus* lectin
HDM	House dust mite
IFN-(type)	Interferon (type)
IFNAR	Interferon-α/β receptor
IgE	Immunoglobin E
IL-(number)	Interleukin-(number)
ILCs	Innate lymphoid cells
infDC	Inflammatory dendritic cell
IntMΦ	Interstitial macrophage
IRF(number)	Interferon regulatory factor (number)
KLF(number)	Kruppel-like factor
LN	Lymph node
LPS	Lipopolysaccharide
*M*. *tuberculosis*	*Mycobacterium tuberculosis*
MAC	Macrophage integrin
Mbd(number)	Methyl-CpG binding domain protein (number)
MelLEC	Clec1a
Mgl2/CD301b	Macrophage galactose N-acetyl-galactosamine specific lectin 2/Cluster of differentiation 301b
moDC	Monocyte-derived dendritic cell
Muc(number)	Mucin (number), oligomeric mucus/gel-forming
MΦ	Macrophage
NFAT	Nuclear factor of activated T cells
NK-kB	Nuclear factor kappa-light-chain-enhancer of activated B cells
NLR	Nod-like receptor
Nlrx(number)	NLR family member X (number)
NOD(number)	Nucleotide-binding oligomerisation domain-containing protein (number)
Nos(number)	Nitric oxide synthase (number)
Nrf(number	Nuclear factor-erythroid factor (number)
OVA	Ovalbumin
OX40L	Tumour necrosis factor receptor superfamily, member 4/OX40 ligand
pDC	Plasmacytoid dendritic cells
PDL(number)	Programmed death ligand (number)
ROS	Reactive oxygen species
SAFS	Severe asthma with fungal sensitisation
scRNA-seq	Single-cell RNA sequencing
SP-(letter)	Surfactant protein (letter)
STAT (number)	Signal transducer and activator of transcription (number)
TAM	Tyro, Axl, MertK receptors
Tfh	T follicular helper cells
TGFβ	Transforming growth factor beta
TLR	Toll-like receptor
TNF	Tumour necrosis factor
TNFR (number)	Tumour necrosis factor receptor (number)
Treg	Regulatory T cell
TSLP	Thymic stromal lymphopoietin
Zeb (number)	Zinc finger e-box binding homeobox (number)

The fungal spores themselves are a crucial aspect in initiating host defence mechanisms. Ungerminated fungal spores are coated with a hydrophobic outer layer of rodlet proteins and melanin upon germination, disruption of this layer reveals numerous fungal motifs on the fungal cell wall (e.g., β-glucan and chitin) that can activate immune responses [24]. If they are not cleared from the airway, spores develop into hyphae secreting numerous components (e.g., glycans, proteases, metabolites, etc.) that aid fungal tissue invasion and can also stimulate immune responses [12]. Mouse models of repeat fungal exposure have shown that spore germination is a crucial factor in the development of allergic inflammatory responses [25,26], demonstrating that fungal motifs are crucial in actively mediating allergic inflammatory responses. Despite this, the role of fungi are less studied in comparison to other allergens such as house dust mite (HDM) [27]. Indeed, murine models of allergic inflammation commonly utilise repeat doses of HDM or use of model antigens in the presence and absence of adjuvants (e.g., OVA and Alum [28]) rather than fungi. Interestingly, fungal components are an underappreciated factor within HDM preparations and can further exacerbate allergic inflammation [29].

Upon sensitisation to allergens, the immune response and resultant cytokine environment mediates many of the features of chronic asthmatic disease [4]. Elevation of type 2 cytokines in the lung (e.g., IL-4, IL-5, and IL-13) is a feature of many asthmatic patients, which orchestrate increases of granulocytes in the airway (e.g., eosinophils and mast cells), activate B cell class switching to IgE and directly activate mucus overproduction, airway hyperresponsiveness and tissue remodelling/fibrosis [28]. However, some asthmatic patients have a lower type 2 response and instead have abundant levels of type 17 cytokines (IL-17 and IL-22) that mediate pathology [30]. Various cell types have been identified as being sources of these cytokines during asthma, including several innate cell populations, e.g., innate lymphoid cells (ILCs), granulocytes, and γδ T cells in addition to adaptive immune cells including CD4^+^ and CD8^+^ T cells [31]. The precise relationship between these responses and the role that MΦ and DCs have in mediating these processes upon fungal exposure are poorly understood, limiting our ability to improve therapeutic strategies.

### Lung macrophages: Promotors or inhibitors of fungal allergic inflammation?

**Fig 1:**
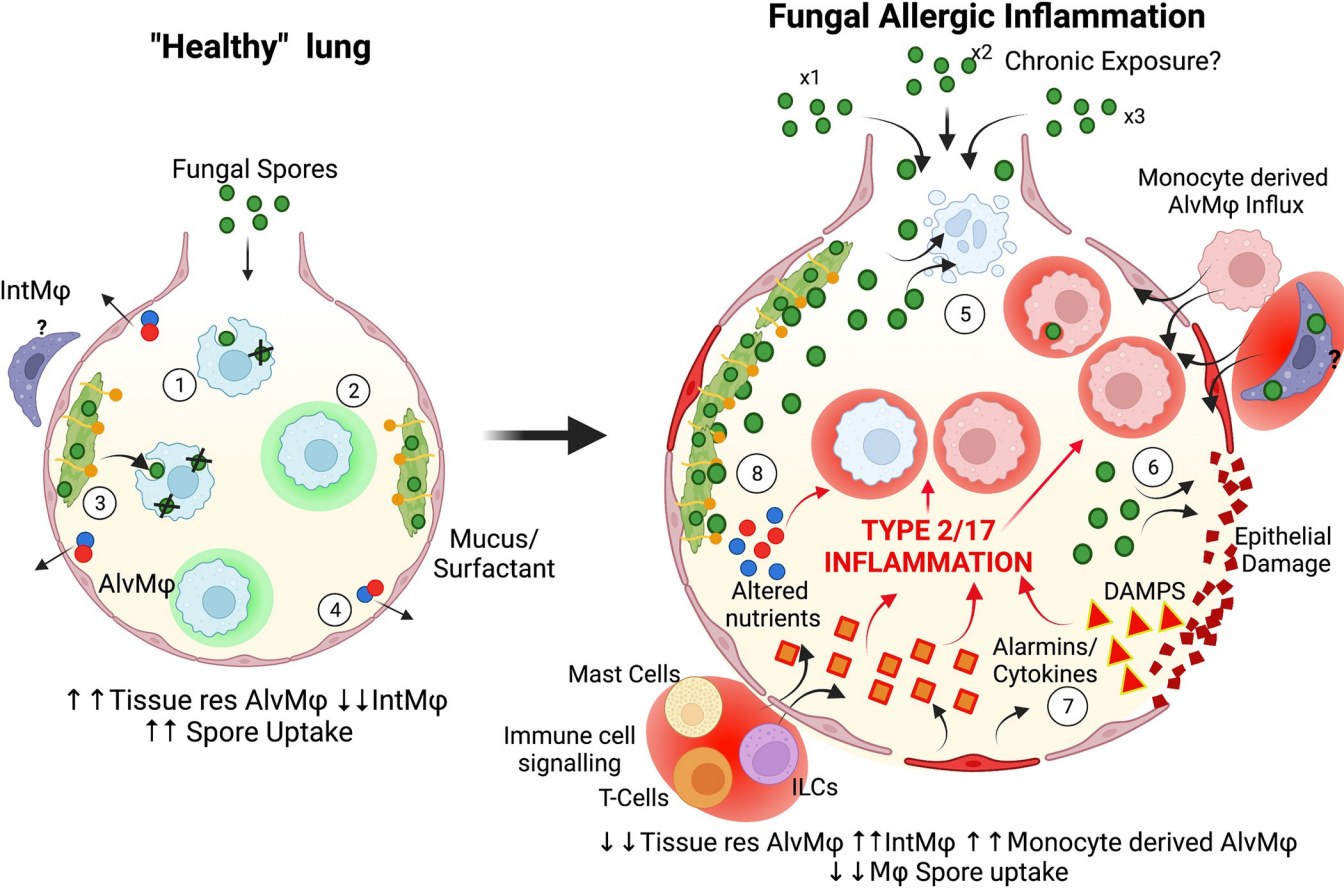
The influence of the lung environment and fungal spores on MΦ responses during allergic inflammation. In a “healthy lung environment” (**left**), (**1**) the majority of inhaled fungal spores are rapidly removed from the airways by AlvMΦ [43]. (**2**) Some AlvMΦ may not acquire spores and generate an anti-inflammatory environment [54]. (**3**) Spore uptake and killing, facilitated by the aid of components of the lung environment including epithelial cell secreted surfactants (SP-A, SP-D) and mucus (particularly the mucin glycoproteins, e.g., Muc5b) [45,159]. (**4**) Other features of the airway include a low nutrient airway environment (maintained by airway epithelial active transporters [170]) that maintain an immunoregulatory MΦ environment [144]. Upon allergic inflammation (**right**), (**5**) repeat spore exposure causes apoptosis or necrosis of resident AlvMΦ [183]. These are replaced by inflammatory IntMΦs [26] or recruited monocytes [37,76]. Both express altered inflammatory transcriptional and epigenetic profiles, leading to differential inflammatory responses upon subsequent spore exposure [71,73]. (**6**) Epithelial cell sensing of fungal material and/or damage to epithelial cell barrier (via fungal proteases), triggers the release of “alarmins” (e.g., TSLP, IL-33, IL-6, IL-22, and CCL2) [173–175]. (**7**) These epithelial signals can recruit and activate other immune cells such as mast cells, basophils and ILC2s inducing a type 2 cytokine environment directly impacting MΦ responses (potentially reducing spore killing) [135–138]. (**8**) Persistence of spores, disrupted epithelial barrier, immune cell infiltration (including CD4^+^ T cells) leads to a type 2 and 17 cytokine environment, alteration of airway nutrient concentrations and hyper secretion of mucus (including Muc5ac) and surfactants [151,158]. These further promote pro-inflammatory MΦ antifungal responses, possibly sustaining allergic inflammation. Figures were created with BioRender.com. AlvMΦ, alveolar macrophage; CCL, chemokine ligand; DAMP, damage-associated molecular pattern; IL, interleukin; IntMΦ, interstitial macrophage; ILC, innate lymphoid cell; MΦ, macrophage; SP, surfactant protein; TSLP, thymic stromal lymphopoietin.

MΦ are widespread throughout the body and are essential for uptake/clearance of foreign pathogens while maintaining tissue homeostasis and development, through clearance of dead cells and debris/particles [32–34]. Upon activation, MΦs are capable of orchestrating downstream effector responses by secreting a wide array of inflammatory mediators (e.g., cytokines and chemokines) and even acting as antigen presenting cells [35]. However, the types of MΦ and their capabilities to elicit inflammatory responses varies depending on their tissue location. In the lung, there are 2 major populations; alveolar MΦs (AlvMΦ) located in the airway (particularly the alveolar sacs) and interstitial MΦs (IntMΦ), which reside within the tissue (Fig 1). These distinctions translate to differences of origin between these MΦ populations [36,37]. AlvMΦ are established by a distinct foetal monocyte population that colonise the lungs rapidly at birth, in steady state conditions these cells self-maintain and comprise the dominant macrophage population in the lung [38]. Conversely, several different populations within the Int MΦ have been identified, the origins of which are still debated, but have been reported to reside in different parts of the lung, e.g., close to lymphatic versus vascular vessels [39,40]. This section will discuss the role of these different macrophage populations in the context of fungal allergic inflammation.

One of the major roles of AlvMΦ populations is maintaining a “healthy” lung environment by removing foreign microbes, particles, and host secreted factors, e.g., MΦs catabolise surfactant secreted by epithelial cells, thus avoiding pulmonary alveolar proteinosis [41,42]. Therefore, AlvMΦ have been proposed to be the dominant cell type that acquires and clears *Af* spores inhaled into the airway [43,44]. There are several reported mechanisms that have been shown to be crucial for this process. Firstly, the spores are able to interact with secretory factors present in the airway which boost MΦ uptake. Melanin on the spore surface interacts with surfactant (particularly surfactant protein D), which boosts macrophage uptake of spores [45]. Furthermore, AlvMΦ express C-type Lectin receptors (CLRs) (e.g., Dectin-1 and 2), which recognise fungal motifs (e.g., β-glucan) revealed on germinating spores, triggering phagocytosis of spores and antifungal immune-based killing [46–48] through phagolysosome acidification and production of reactive oxygen species (ROS) [43,49].

While in health AlvMΦ clear spores without eliciting significant inflammatory responses, they can also mediate significant downstream antifungal pro-inflammatory responses, by secreting large amounts of cytokines/chemokines (e.g., IL-1α, IL-1β, IL-6, and TNFα) upon activation of CLR (e.g., Dectin-1), Toll-like receptor (TLR) (e.g., TLR4), and inflammasome signalling pathways [50–53]. The precise factors that govern whether AlvMΦs balance spore clearance, with minimal inflammation or significant inflammatory responses when required remains unclear. Recent evidence has shed some light by showing that *Af* spores can elicit differential AlvMΦ responses, measured through secretion of CXCL2 (a neutrophil chemoattractant). This heterogeneity has functional relevance as CXCL2^+^ AlvMΦs were the dominant population that acquired spores and exhibited higher levels of metabolic activity, compared to CXCL2^−^ counterparts which displayed a more anti-inflammatory profile (characterised by expression of IL-10 and complement C1q component) [54]. The authors also observed plasticity between these AlvMΦ subsets, as instillation of bacterial ligands pushed all AlvMΦ towards a CXCL2^+^ phenotype. This heterogeneity of AlvMΦ responses to fungi, and the impact on allergic inflammation upon frequent exposure to *Af* spores, is an important question for future studies.

In their steady-state role, AlvMΦs can also induce regulatory T cells (Treg) generating a regulatory cytokine milieu (e.g., IL-10 and TGFβ) in the lung [55–59] (Fig 1). This has been attributed as preventing, rather than promoting, the development of allergic inflammatory disease [60,61]. For example, in murine asthma models, depletion of AlvMΦ (via clodronate liposomes) exacerbated inflammation, while adoptive transfer of AlvMΦ from naive mice reduced airway hyperresponsiveness [62]. In contrast, others suggest a role for AlvMΦs in contributing to the development of allergic inflammation via pathogenic IL-17 signalling, as well as hypersecretion of pro-inflammatory cytokines (TNF, IL-6, IFN-β, and CXCL2) [56,63]. These conflicting results could reflect functional heterogeneity of lung AlvMΦs, and divergent outcomes are dependent on the context and timing of allergen exposure. Surprisingly, given its importance in anti-spore responses, it is unclear whether AlvMΦ CLR-signalling is important in triggering allergic inflammation. Studies have suggested that Dectin1^−/−^ mice have disrupted allergic inflammation in response to *Af* spores, although the relative role of MΦs was not assessed [64]. In contrast, TLR signalling on lung MΦs has been proposed to instigate allergic inflammatory responses against spores. Fungal protease cleavage of host fibrinogen (generating fibrinogen cleavage products, FCPs) activate MΦ via TLR4 and the macrophage integrin (Mac-1), boosting macrophage fungistatic responses and triggering allergic inflammation [65,66]. These FCPs can also activate other cell types such as epithelial cells, mast cells, and DCs [67,68]. While it is clear that AlvMΦs are crucial for spore clearance, much remains unknown about how this role changes, and the relative contribution of AlvMΦ in development of allergic inflammation against fungi.

The role of IntMΦ, in mediating allergic inflammatory responses to inhaled fungi, is largely unexplored. In the context of bacterial lung infection and lung fibrosis, IntMΦ have been suggested to exhibit both pro- and anti-inflammatory capabilities [69]. A recent study utilised single-cell RNA sequencing (scRNA-seq) on lung MΦs from mice infected with transgenic *M*. *tuberculosis* to identify the fitness of the bacterial cells inside the MΦ population. This revealed 3 IntMΦ populations induce different bacterial responses; a monocyte origin MΦ subset (identified via Nos2) induced bacterial stress responses, and anti-inflammatory MΦ (expressing Nrf2) subset caused bacterial sensing of environmental stress and a Zeb2-expressing MΦ subset appear to be involved in resolving inflammation [70]. Whether these IntMΦ populations are present and mediating similar responses in the lung following fungal exposure is an important point to address with future studies. When considering MΦ responses in the lung, it is important to reflect that upon inflammation, the AlvMΦ and IntMΦ tissue niches can be repopulated with MΦ of monocyte origin with markedly altered functional capabilities [26] (Fig 1). In the context of bacterial infection or viral infection during asthma, the replacement of AlvMΦs with monocyte-derived AlvMΦs resulted in markedly altered function, with impaired phagocytosis and responsiveness reducing allergic inflammation [71,72]. Conversely, murine asthma models have demonstrated monocyte-derived AlvMΦs display a higher inflammatory potential, driving development of allergic inflammation [60,73]. This suggests monocyte replacement of AlvMΦs could be heavily influenced by the inflammatory environment of the lung. For example, LPS has been found to expand IL-10 secretion of IntMΦs reducing DC-mediated induction of allergic responses [74]. In the context of invasive aspergillosis, recruitment of CCR2^+^ monocytes have been shown to be crucial for orchestrating clearance of fungal spores [75,76]. The relative role of monocytes in replacing MΦ populations and the potential impact this has in the context of fungal allergic inflammation remains unclear.

### The role of dendritic cells in mediating, sustaining, and dampening fungal allergic inflammation

DCs, which bridge innate and adaptive immune responses, are essential in eliciting, sustaining, and dampening lung allergic inflammation [10,77]. In the lung, DCs acquire potential allergens and migrate to the draining lymph nodes (LNs) activating antigen specific T cell responses [78,79]. However, DCs can also be “tolerogenic” and halt the progression of allergic inflammation, predominately via promoting Tregs [80]. In the context of anti-fungal allergic inflammation, earlier literature suggested that differential uptake of *Af* conidia versus hyphae mediates DCs to elicit type 1 (IFNγ mediated) anti-fungal immunity or type 2 associated allergic inflammation, respectively [81]. Also it has been suggested that fungal exposure can cause DCs to dampen allergic inflammation by driving tolerogenic responses [82]. Yet, the precise mechanisms that DCs employ to initiate and/or dampen chronic fungal allergic inflammation are poorly understood. This is partially due to the fact that the DC population is heterogeneous, consisting of multiple separate subsets and each with differing functional capabilities. It has proved technically challenging to definitively identify these subsets, making manipulation of these different populations difficult. This section will explore the role that different DC subsets have in mediating antifungal immunity and chronic allergic inflammation.

**Fig 2:**
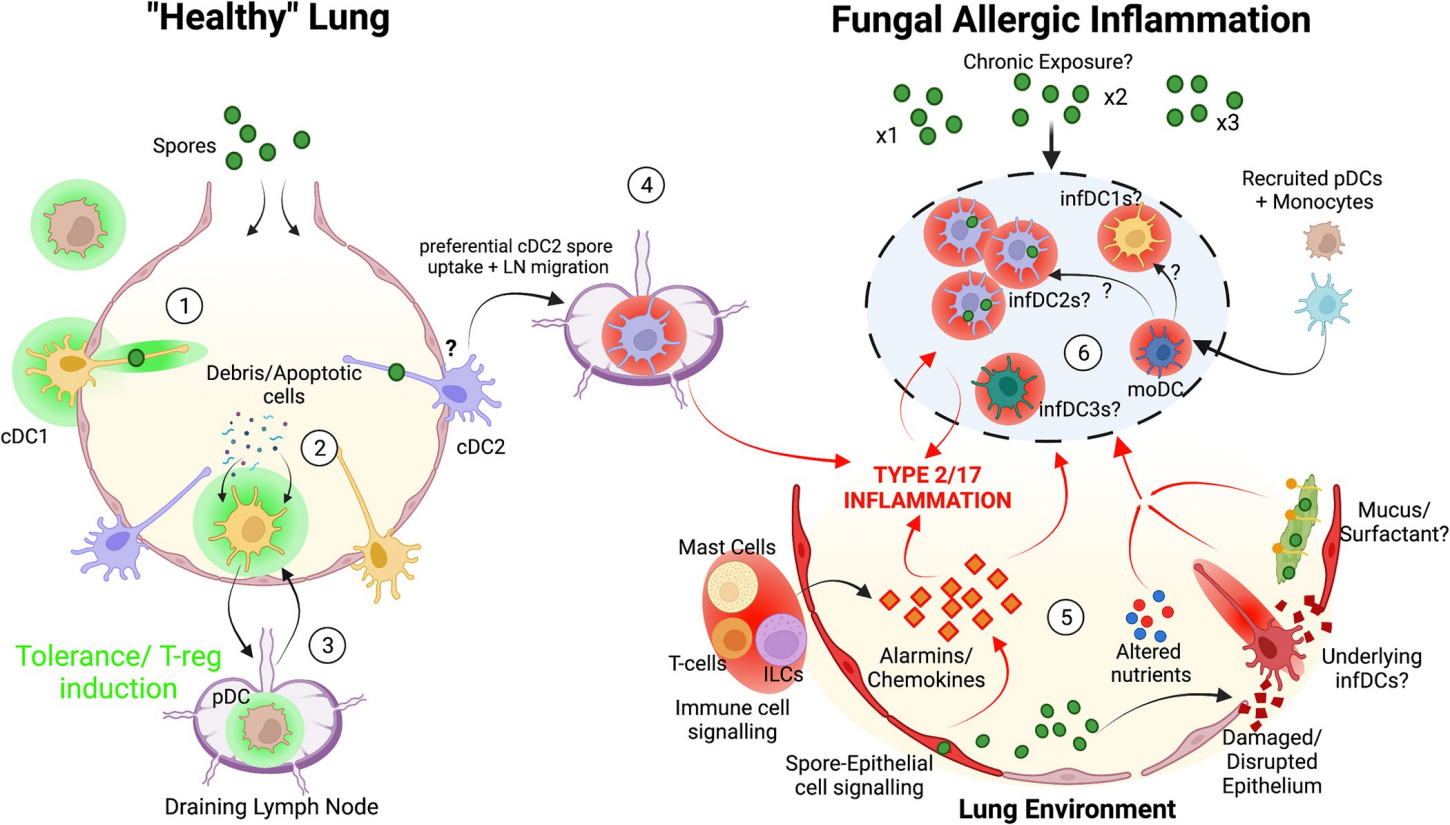
Understanding how DC induction of fungal allergic inflammation is shaped by the lung environment. In health (**left**), DCs predominantly reside in the tissue but can project dendrites into the airway to sample antigen. (**1**) As AlvMΦs predominantly clear inhaled spores [43], exposure of DCs to fungal antigen is minimal reducing potential for inflammatory responses. (**2**) DC subsets, especially cDC1s, assume housekeeping duties (e.g., clearance of apoptotic cells) maintaining a tolerogenic phenotype. (**3**) Upon migration to draining LN lung DCs, in concert with other subsets such as pDCs, induce T-reg generation further maintaining an immuno-regulatory lung environment. (**4**) Fungal allergic inflammation is initiated upon cDC2 acquisition of *Af* spores and migration to the draining LN where they can prime adaptive CD4^+^ T cell responses (**right**). (**5**) While the precise mechanisms by which cDC2 mediate these responses to spores is unclear, the lung environment is known to directly influence this process. Fungal secretory products (including proteases) in the airway lumen can not only activate DCs directly, but also damage the epithelial barrier. This allows spores to move beyond the epithelial barrier and potentially activate cDC2s in the deeper underlying tissue. Furthermore, epithelial cell responses to fungi and/or barrier damage triggers the release of alarmins, chemokines, cytokines, and DAMPs (e.g., CCL2, IL-6, IL-33, and TSLP^173,^ [174,175]), which can further activate DCs to promote allergic response. In addition, ILCs and mast cells (which can be activated by epithelial signals) further promote type 2 and type 17 cytokine which further conditions DCs to exacerbate allergic inflammation [68,145,180,184]. Other lung environmental factors such as altered nutrient availability and increased surfactant/ mucus concentrations [45,158] can further shape DC responses. (**6**) These features can lead to the formation of several inflammatory DC states (infDC1, infDC2, and infDC3) and possibly DCs differentiated from monocytes (moDCs) which further amplify and sustain fungal allergic inflammatory disease. Figures were created with BioRender.com. AlvMΦ, alveolar macrophage; CCL, chemokine ligand; DC, dendritic cell; IL, interleukin; moDC, monocyte-derived dendritic cell TSLP, thymic stromal lymphopoietin.

Broadly, DCs are grouped into 2 major DC subsets, conventional DCs (cDCs) and plasmacytoid DCs (pDCs) [83]. Based on differences in development, marker expression and functional capabilities, cDCs can be further classified as cDC1s (dependent on BATF3 and IRF8) or cDC2s (dependent on IRF4 and KLF4) [84,85]. Lung resident cDC1s are potent at mediating CD8^+^ T cell activation via cross presentation [86] and type 1 CD4^+^ T cell responses against viral and bacterial pathogens [87,88]. In comparison, cDC2s have been proposed to directly mediate type 2 and type 17 CD4^+^ T cell responses to a range of pathogens (including helminth parasites, fungi, and bacteria) [89,90]. Understanding the role of these subsets in inflammatory environments has proven challenging. For example during allergic inflammation, cDC2s can adopt an “inflammatory-like profile” (infDC2) and contribute to antiviral type 1 responses [91]. Others have proposed a presence of an “inflammatory” DC3 subset, which do not appear to express traditional markers of cDC1 and cDC2 cells but potentially can induce different types of T cell responses [92]. In addition to cDC subsets, recruited monocytes have been reported to develop into monocyte-derived DCs (moDCs) with capabilities of mediating inflammatory responses [93] (Fig 2). The complexity of accurately defining these subsets has made it difficult to understand the relative roles of these varying subsets in allergic inflammation.

#### Context-dependent role of pDCs in allergic inflammation

While pDCs are crucial for anti-viral immunity, they have also been proposed to have a protective role during invasive fungal disease [94]. CLR expression on human pDCs (e.g., Dectin-2) enables them to recognise *Af* and suppress hyphal growth through secretion of protective pro-inflammatory cytokines (IL-12, TNF-α, and IFN-α [95,96]) and release of extracellular traps [97]. This is underlined with a recent study that showed in response to *Af* spores, recruitment of pDCs via CXCL9 and CXCL10 enhances neutrophil spore killing [94].

On the observations in HDM- and OVA-induced asthma models, pDCs have been reported to dampen allergic inflammation (utilising depletion and cell transfer strategies) [98–100]. In the context of fungi, transfer of pDCs from *Af* sensitised mice successfully suppressed allergic inflammation via IL-10 secretion in recipient mice [101]. Furthermore, pDCs can mediate Treg generation leading to dampening of airway hyperreactivity [102]. In contrast, other studies have observed that pDC may exacerbate allergic inflammation [103]. For example, complement C3a component reduces pDC expression of PDL1 and PDL2 leading to the promotion of fungal allergic inflammation [104]. These discrepancies may suggest that the timing of pDC recruitment and activation, as well as subsequent signals from the lung environment upon their arrival govern their ability to direct fungal allergic disease.

#### Are cDC1s important in fungal allergic inflammation?

The cDC1 subset is crucial for initiating type 1 protective immune responses (e.g., targeting pathogens and cancer) and tissue homeostasis, via uptake and clearance of apoptotic cell antigens (e.g., via the CLR and DNGR1) [105,106]. In the context of allergic inflammation, the majority of studies suggest that cDC1s appear to dampen, rather than initiate, these responses [10,92] (Fig 2). This is based on the fact that cDC1 deficient mice (e.g., CD103^−/−^ and *Batf3*^−/−^) mount greater allergic inflammation in both OVA and HDM based models [107,108]. This restraining allergic airway inflammation is mediated via cDC1 secretion of IL12 limiting type 2 inflammation [107].

In the context of fungal infection, there is limited research into the potential role of cDC1s in shaping allergic inflammation. Upon invasive fungal disease, cDC1s secrete IL-2 upon recognition of germinated fungi via the Ca2^+^ calcineurin-NFAT pathway which is crucial for protective (not pathogenic) type 17 responses [109]. A recent study has highlighted that cDC1s expression of Nlrx1 (NOD9, a negative regulator of downstream NK-kB–mediated responses) limits ability to induce type 2 inflammation during invasive fungal disease [110]. In the context of other fungi, cDC1s can be dispensable (e.g., against *Candida albicans* in the intestine) [111] or essential (e.g., mediating type 1 protective responses to the dimorphic fungus *Histoplasma*) [112]. Therefore, these studies suggest that cDC1 have the potential to play some role in shaping type 1, 2, and 17 anti-*Af* responses (Fig 2). However, more investigations are needed to define the specific role of pulmonary cDC1s in a setting of chronic exposure to fungal spores and the ensuing allergic inflammation.

#### The role of cDC2 subsets in fungal allergic inflammation

Numerous studies have highlighted that cDC2s are crucial in mediating allergic inflammation [85,89]. Indeed, they are the major DC subset to acquire allergens from the airway (following HDM or OVA administration) and subsequently migrate to draining LNs [88,89]. Upon arrival, cDC2 can mediate type 2 [85,89], type 17 [113,114], and follicular (Tfh) CD4^+^ T cells [115] responses to allergens (e.g., HDM). This was established via transfers of cDC2s and use of cDC2 deficient mice (*Irf4*^fl/fl^*Cd11c*^cre^ mice [85,116]) (Fig 2). In addition to the resident cDC2s during sensitisation, repeated allergen exposure can also mediate significant expansion and/or recruitment of lung cDC2s [117]. In the context of fungi elicited allergy, similar to MΦs, DCs (likely cDC2s, although not determined in study) have been shown to respond to FCPs and type 2 cytokine (IL-13) via up-regulation of PDL2, boosting their ability to mediate type 2 inflammation [68]). Interestingly, cDC2 were found to be crucial in mediating protective type 2 responses against *Cryptococcus* infection [118], while cDC2s have also been identified in eliciting protective type 17 responses in response to invasive *Af* infection [90]. Whether the same cDC2 population is important in driving over exuberant type 17 inflammation, in addition to type 2 responses, to fungi during allergic inflammation has not been fully explored.

Despite a demonstrated role for cDC2s in mediating allergic inflammation, the mechanism(s) that they utilise to orchestrate downstream inflammation is unclear. A range of cell surface molecules (e.g., CD40, CD86, Dectin-2, IFNAR, Mgl2, OX40L, PDL1, and PDL2), intracellular mediators (e.g., Mbd2 and Stat5) and secreted cytokines and chemokines (e.g., IL-10, IL-33, CCL17, and CCL22) have been suggested [10,68,113,119,120]. In particular, a recent study proposed that cDC2s expression of IFNAR1 and TNFR2 enables them to generate Tregs in steady state conditions and type 2 responses upon HDM challenge [121]. Further work suggested that IFNβ signalling can render cDC2s tolerogenic, ameliorating HDM allergic inflammation [122]. This suggests that, similar to the other DC subsets, the timing of stimuli may influence the mechanisms that cDC2 employ to mediate allergic inflammation.

In addition to cDC2s, moDCs (defined as CD64^+^FcεR1^+^) have been proposed to be important to induce pulmonary allergic inflammation. This was shown as moDCs were able to initiate allergic inflammation in the absence of lung cDC subsets [89]. Additionally, transfer of moDCs induced type 2 allergic inflammation [123], indicating moDCs are important to induce pulmonary allergic inflammation Others have proposed that moDCs are the main mediators of the “effector” stage of the allergic response by producing the chemokine milieu responsible for recruiting eosinophils, effector T cells and mononuclear cells (via secretion of CCL2, CCL4, CCL9, and CCL24) to the lungs [89]. In response to invasive disease, moDCs have been reported to mediate fungal killing as well as secreting TNF and IL12p70 stimulating neutrophil-mediated fungal clearance [75]. Also, moDC secretion of CXCL9/10 appears important to the recruitment of pDCs, with this crosstalk crucial in mediating immunity to invasive aspergillus infection [124]. Importantly, moDC secretion of TNFα has been proposed to mediate type 17 inflammation following chronic *Af* exposure [125]. This suggests that moDCs may play a crucial role either directly, or in collaboration with other DC subsets, to mediate fungal allergic inflammation (Fig 2). However, when considering the potential role of moDCs, it is important to reflect on recent studies that have identified previously unrecognised subsets like infDC2s and DC3s [126,127]. Indeed, formation of these subsets are likely dependent on the inflammatory context [91,128,129]. Definitively, separating these populations from cDC2 and moDC subsets is challenging. Indeed, scRNA-seq studies suggest that previous strategies to identify moDCs actually contain infDC2s that also express higher levels of CCR2 [91,130] and its these and not “moDCs” that mediate allergic inflammation^91^. Therefore, the relative role for infDC2s, DC3s and moDCs, and the mechanism(s) they employ in mediating fungal allergic inflammation is an important question for future studies to tackle.

### How the lung environment governs myeloid cells in mediating fungal allergic disease

It has become clear that tissue microenvironments are critical in shaping the development and functional capacity of MΦs and DCs. Indeed, the role of the lung environment on shaping MΦ function has been well explored [34,37,131,132], and recent work is now underlining the importance of the environmental influence on shaping DC responses [36,133]. Moreover, many aspects of the lung environment change during chronic lung inflammation, and it is important to consider the differing impacts these may have on governing how MΦs and DCs mediate antifungal allergic disease.

#### Alteration of secretory mediators in the lung environment

One of the major changes in the lung environment upon the onset of allergic inflammation is the increase in type 2 cytokines. These can trigger “alternative” M(IL-4) activation of MΦ, associated with enhancing fibrosis through aberrant wound repair responses [134]. These MΦ display elevated expression of arginase-1 (diverting L-arginine metabolism away from nitric oxide production) and chitinase-like proteins [135–138]. In addition to type 2 cytokine, others have suggested that surfactant protein A, uptake of apoptotic cells via TAM receptors, and chitin (a crucial constituent of the fungal cell wall) can mediate M(IL-4) activity [139–141] (Fig 1). The functional impact of these M(IL-4) MΦ on fungal allergic inflammation is unclear, but it has been proposed to boost MΦ ability to clear *Af* spores while others have suggested these exacerbate responses [142,143]. Furthermore, AlvMΦ in the lung airway are less able to respond to type 2 cytokine compared to IntMΦ, which reside in the tissue [144]. The impact of these environmental cytokine and fungal signals on MΦ subset function during allergic inflammation is unknown.

Type 2 cytokine signals are also known to be crucial in shaping DC maturation and functional capabilities (Fig 2), e.g., IL-13 and IL-33 released by ILC2s has been proposed to enhance cDC2 generation of type 2 responses in the lung and skin [145,146]. A recent study has further highlighted this by demonstrating that IL-13 in the skin environment shapes cDC2s to mediate type 2 responses, and if absent DCs, elicit a type 17 response instead [147]. The impact that differing lung cytokine environments, induced during allergic inflammation, have on governing DC subset development and capacity to respond in the context of antifungal inflammation is an important question for further research.

Another critical change to the lung environment during allergic inflammation is increased secretion of mucus and surfactant into the airway [148–154]. Indeed, mucus plugging is prominent in cases of severe asthma [155,156]. A major constituent of mucus are polymeric mucin glycoproteins (e.g., Muc5b and Muc5ac) that can directly interact with immune cells as evident by the fact that Muc5b-deficient mice are susceptible to bacterial infection due to impaired MΦ responses [157]. Furthermore, Muc5ac has been proposed to be important for mediating allergic airway hyperreactivity against *Af* extract [158]. Strikingly, FleA protein expression on *Af* spore surface readily binds with mucin glycoproteins enhancing MΦ spore uptake [159], while surfactant protein D, (elevated in allergic diseases) boosts fungal spore uptake by MΦs [45] (Fig 1). Recent work demonstrates that intestinal mucin proteins (Muc2) shape DC activation and cDC2 development [160,161] (Fig 2). Additionally, seminal work has shown that spontaneous protein crystallisation (Charcot–Leyden crystals, CLCs), which can form in the airways of asthma patients, have the potential to drive cDC2s to mediate allergic inflammatory responses [162]. The relative role of mucus and surfactant in shaping MΦ and DC allergic inflammation in response to fungi remains poorly understood.

#### Metabolic activity within the lung environment

The metabolic state of MΦ and DC populations greatly influences their functional capabilities. Both cell types can utilise distinct metabolic pathways for energy production which governs their downstream activity, impacting chronic lung disease [163,164]. For example, tolerogenic DCs and M(IL-4) MΦs rely on mitochondrial respiratory chain and fatty acid oxidation, whereas inflammatory DCs and MΦs rapidly up-regulate glycolytic activity [163,165,166]. Indeed, fungal stimulation of both MΦ and DCs can lead to a rapid transition from utilising one metabolic pathway to another (e.g., from fatty acid oxidation to glycolysis) as the main energy source for cellular activity [167–169]. This can also be regulated by the tissue environment, with AlvMΦs or transferred MΦs that reside in the airway exhibiting dampened glycolytic activity reducing their potential to respond to type 2 inflammation [144]. While the precise factors in the airway that cause this are unclear, an important aspect could be the amount and/or type of nutrients in the lung which are altered in many chronic inflammatory lung disease [170]. Therefore, DC and MΦ metabolic activity that is possibly regulated by nutrient availability maybe critical in governing the downstream fungal allergic inflammation (Figs 1 and 2).

#### Lung epithelial and innate cell crosstalk

The airway epithelial barrier itself has a crucial role in governing MΦ and DCs responses [10]. Proteases secreted from germinating *Af* spores (e.g., Aspf13 and Alp-1) disrupts the epithelial barrier, increasing permeability [171,172]. This enables fungal allergens to cross the disrupted epithelial barrier into the lung tissue and stimulates calcium flux (via calcineurin) within epithelial cells further activating DCs and IntMΦs [173]. This suggests that fungi are more likely to be exposed to pro-inflammatory cells (e.g., IntMΦ and inflammatory DCs) rather than normal regulatory AlvMΦs and DCs, which reside in the airway, and may trigger and sustain allergic inflammation (Figs 1 and 2). In addition to this, epithelial cells can release various pro-allergy mediators such as IL-33, TSLP, IL-17, IL-6, IL-8, IL-25, and CCL2 [73,174–176] and damage-associated molecules such as uric acid, calcium, and calcineurin [173,177], all of which facilitates crosstalk that can trigger the activation of lung resident MΦ [73,178] and DCs [146,179] to promote allergic inflammatory responses (Figs 1 and 2). These epithelial-mediated signals (e.g., IL-33) can boost ILC2-mediated responses, leading to the secretion of type 2 cytokines and triggering both MΦ and DC to induce allergic inflammation [180,181] (Figs 1 and 2). Despite the evidence of epithelial crosstalk with lung MΦs, DCs and ILCs, the relative importance of these interactions in governing fungal allergic inflammation is yet to be fully explored. In addition to epithelial cells, endothelial cell recognition of *Af*, via the CLR MelLEC, has been shown to promote allergic inflammation. Although what impact endothelial cell recognition of spores has on MΦ and DC induction of fungal allergic inflammation is unclear. Fungal material can also promote a type 2 cytokine environment by inducing mast cells to secrete IL-13 [68] and activated mast cells can trigger AlvMΦs to promote allergic inflammation [63]. Therefore, it is clear that numerous cell types in the lung can “interact” with MΦ and DC populations and alter downstream inflammatory responses in response to fungi. Yet, in order to build an accurate model of on the pathogenesis of allergic bronchopulmonary mycoses, further work is needed to understand which of these cellular interactions are critical in governing MΦ and DC antifungal activity.

## Concluding remarks

In summary, the recent advances in single cell approaches have resulted in vast improvements in our understanding of how MΦ and DC subsets govern inflammation that underpins allergic disease. This review has discussed the roles of MΦ and DC subsets in fungal allergic inflammation and highlighted several areas where our current understanding is limited. Future important questions remain unanswered. For example, this review has mainly considered the impact of *Af* spore exposure only on MΦ and DC responses. Whereas in the majority of cases, individuals will be exposed to *Af* in combination with other well-known allergens (e.g., HDM) and even other fungi which can promote allergic inflammation [182]. Understanding this complexity and defining the dominant allergen signals could greatly inform future diagnostic approaches. Finally, in addition to considering the host lung environmental factors we highlighted, it is clear that the micro- and myco-biome in the airways and distal sites can profoundly influence immune responses (e.g., the gut–lung and skin–lung axis). How these wider diverse microbial interactions fit with intrinsic cues and epithelial innate immune cell crosstalk in the lung microenvironment, and how they together influence MΦ and DC responses upon fungal spore exposure, is an additional challenge for future research. Ultimately a better understanding of how MΦs and DCs respond upon fungal exposure in the wider context of the lung environment may yield novel therapeutic strategies to combat the growing problem of fungal allergic disease.

## References

[1] Peay KG, Kennedy PG, Talbot JM. Dimensions of biodiversity in the Earth mycobiome. Nat Rev Microbiol 2016 147. 2016;14: 434–447. doi: 10.1038/nrmicro.2016.59 27296482

[2] Agarwal R, Chakrabarti A, Shah A, Gupta D, Meis JF, Guleria R, et al. Allergic bronchopulmonary aspergillosis: review of literature and proposal of new diagnostic and classification criteria. Clin Exp Allergy. 2013;43:850–73. doi: 10.1111/cea.12141 23889240

[3] Knutsen AP, Bush RK, Demain JG, Denning DW, Dixit A, Fairs A, et al. Fungi and allergic lower respiratory tract diseases. J Allergy Clin Immunol. 2012;129:280–91. doi: 10.1016/j.jaci.2011.12.970 22284927

[4] Rick EM, Woolnough K, Pashley CH, Wardlaw AJ. Allergic fungal airway disease. J Investig Allergol Clin Immunol. 2016;26:344–54. doi: 10.18176/jiaci.0122 27996940

[5] Masaki K, Fukunaga K, Matsusaka M, Kabata H, Tanosaki T, Mochimaru T, et al. Characteristics of severe asthma with fungal sensitization. Ann Allergy Asthma Immunol. 2017;119:253–7. doi: 10.1016/j.anai.2017.07.008 28801088

[6] Agarwal R, Gupta D. Severe asthma and fungi: current evidence. Med Mycol. 2011;49:S150–7. doi: 10.3109/13693786.2010.504752 20662637

[7] Denning DW, Pashley C, Hartl D, Wardlaw A, Godet C, Del Giacco S, et al. Fungal allergy in asthma–state of the art and research needs. Clin Transl Allergy. 2014;4:14. doi: 10.1186/2045-7022-4-14 24735832 PMC4005466

[8] Denning DW O’Driscoll BR, Hogaboam CM, Bowyer P, Niven RM. The link between fungi and severe asthma: a summary of the evidence. Eur Respir J. 2006;27:615–26. doi: 10.1183/09031936.06.00074705 16507864

[9] Robbe P, Draijer C, Borg TR, Luinge M, Timens W, Wouters IM, et al. Distinct macrophage phenotypes in allergic and nonallergic lung inflammation. Am J Physiol Lung Cell Mol Physiol. 2015;308:L358–467. doi: 10.1152/ajplung.00341.2014 25502502

[10] Lambrecht BN, Hammad H. The role of dendritic and epithelial cells as master regulators of allergic airway inflammation. Lancet. 2010;376:835–43. doi: 10.1016/S0140-6736(10)61226-3 20816550

[11] Fricker M, Gibson PG. Macrophage dysfunction in the pathogenesis and treatment of asthma. Eur Respir J. 2017;50:1700196. doi: 10.1183/13993003.00196-2017 28899935

[12] Bartemes KR, Kita H. Innate and adaptive immune responses to fungi in the airway. J Allergy Clin Immunol. 2018;142:353–63. doi: 10.1016/j.jaci.2018.06.015 30080527 PMC6083885

[13] Romani L. Immunity to fungal infections. Nat Rev Immunol 2011 114. 2011;11: 275–288. doi: 10.1038/nri2939 21394104

[14] Agarwal R. Severe asthma with fungal sensitization. Curr Allergy Asthma Rep. 2011;11:403–13. doi: 10.1007/s11882-011-0217-4 21789577

[15] Moss RB. Pathophysiology and immunology of allergic bronchopulmonary aspergillosis. Med Mycol. 2005;43:S203–6. doi: 10.1080/13693780500052255 16110813

[16] Masoli M, Fabian D, Holt S, Beasley R. The global burden of asthma: executive summary of the GINA Dissemination Committee Report. Allergy. 2004;59:469–78. doi: 10.1111/j.1398-9995.2004.00526.x 15080825

[17] Dharmage SC, Perret JL, Custovic A. Epidemiology of asthma in children and adults. Front Pediatr. 2019;7:246. doi: 10.3389/fped.2019.00246 31275909 PMC6591438

[18] Simon-Nobbe B, Denk U, Pöll V, Rid R, Breitenbach M. The Spectrum of Fungal Allergy. Int Arch Allergy Immunol. 2008;145:58–86. doi: 10.1159/000107578 17709917

[19] Lacey J. Spore dispersal—Its role in ecology and disease: The British contribution to fungal aerobiology. Mycol Res. 1996;100:641–60. doi: 10.1016/S0953-7562(96)80194-8

[20] Guinea J, Peláez T, Alcalá L, Bouza E. Outdoor environmental levels of Aspergillus spp. conidia over a wide geographical area. Med Mycol. 2006;44:349–56. doi: 10.1080/13693780500488939 16772229

[21] De Gómez AS, Torres-Rodríguez JM, Alvarado Ramírez E, Mojal García S, Belmonte-Soler J. Seasonal distribution of Alternaria, Aspergillus, Cladosporium and Penicillium species isolated in homes of fungal allergic patients. J Investig Allergol Clin Immunol. 2006;16:357–63. Available: https://europepmc.org/article/med/17153883. 17153883

[22] Kosmidis C, Denning DW. The clinical spectrum of pulmonary aspergillosis. Thorax. 2015;70:270–7. doi: 10.1136/thoraxjnl-2014-206291 25354514

[23] Denning DW. Invasive aspergillosis. Clin Infect Dis. 1998;26:781–805. doi: 10.1086/513943 9564455

[24] Van De Veerdonk FL, Gresnigt MS, Romani L, Netea MG, Latgé JP. Aspergillus fumigatus morphology and dynamic host interactions. Nat Rev Microbiol. Nature Publishing Group; 2017. pp. 661–674. doi: 10.1038/nrmicro.2017.90 28919635

[25] Murdock BJ, Shreiner AB, McDonald RA, Osterholzer JJ, White ES, Toews GB, et al. Coevolution of TH1, TH2, and TH17 responses during repeated pulmonary exposure to aspergillus fumigatus conidia. Infect Immun. 2011;79:125–35. doi: 10.1128/IAI.00508-10 21041495 PMC3019910

[26] Dietschmann A, Schruefer S, Krappmann S, Voehringer D. Th2 cells promote eosinophil-independent pathology in a murine model of allergic bronchopulmonary aspergillosis. Eur J Immunol. 2020;50:1044–56. doi: 10.1002/eji.201948411 32108934

[27] Crameri R, Garbani M, Rhyner C, Huitema C. Fungi: the neglected allergenic sources. Allergy. 2014;69:176–85. doi: 10.1111/all.12325 24286281

[28] Hammad H, Lambrecht BN. The basic immunology of asthma. Cell Elsevier. 2021:1469–85. doi: 10.1016/j.cell.2021.02.016 33711259

[29] Hadebe S, Kirstein F, Fierens K, Redelinghuys P, Murray GI, Williams DL, et al. β-Glucan exacerbates allergic airway responses to house dust mite allergen. Respir Res. 2016;17:1–3. doi: 10.1186/s12931-016-0352-5 27039089 PMC4818888

[30] De Luca A, Pariano M, Cellini B, Costantini C, Villella VR, Jose SS, et al. The IL-17F/IL-17RC Axis Promotes Respiratory Allergy in the Proximal Airways. Cell Rep. 2017;20:1667–80. doi: 10.1016/j.celrep.2017.07.063 28813677

[31] Lambrecht BN, Hammad H. The immunology of asthma. Nat Immunol 2014 161. 2014;16:45–56. doi: 10.1038/ni.3049 25521684

[32] Westphalen K, Gusarova GA, Islam MN, Subramanian M, Cohen TS, Prince AS, et al. Sessile alveolar macrophages modulate immunity through connexin 43-based epithelial communication. Nature. 2014;506:503. doi: 10.1038/nature12902 24463523 PMC4117212

[33] Han CZ, Juncadella IJ, Kinchen JM, Buckley MW, Klibanov AL, Dryden K, et al. Macrophages redirect phagocytosis by non-professional phagocytes and influence inflammation. Nat 2016 5397630. 2016;539:570–574. doi: 10.1038/nature20141 27820945 PMC5799085

[34] Hussell T, Bell TJ. Alveolar macrophages: plasticity in a tissue-specific context. Nat Rev Immunol 2014 142. 2014;14:81–93. doi: 10.1038/nri3600 24445666

[35] Muntjewerff EM, Meesters LD, van den Bogaart G. Antigen Cross-Presentation by Macrophages. Front Immunol. 2020;11:1276. doi: 10.3389/fimmu.2020.01276 32733446 PMC7360722

[36] Guilliams M, Svedberg FR. Does tissue imprinting restrict macrophage plasticity? Nat Immunol 2021 222. 2021;22:118–127. doi: 10.1038/s41590-020-00849-2 33462453

[37] Bain CC, MacDonald AS. The impact of the lung environment on macrophage development, activation and function: diversity in the face of adversity. Mucosal Immunol. 2022;2022:1–12. doi: 10.1038/s41385-021-00480-w 35017701 PMC8749355

[38] Guilliams M, De Kleer I, Henri S, Post S, Vanhoutte L, De Prijck S, et al. Alveolar macrophages develop from fetal monocytes that differentiate into long-lived cells in the first week of life via GM-CSF. J Exp Med. 2013;210:1977–92. doi: 10.1084/jem.20131199 24043763 PMC3782041

[39] Gibbings SL, Thomas SM, Atif SM, McCubbrey AL, Desch AN, Danhorn T, et al. Three unique interstitial macrophages in the murine lung at steady state. Am J Respir Cell Mol Biol. 2017;57:66–76. doi: 10.1165/rcmb.2016-0361OC 28257233 PMC5516280

[40] Schyns J, Bai Q, Ruscitti C, Radermecker C, De Schepper S, Chakarov S, et al. Non-classical tissue monocytes and two functionally distinct populations of interstitial macrophages populate the mouse lung. Nat Commun 2019 101. 2019;10:1–16. doi: 10.1038/s41467-019-11843-0 31481690 PMC6722135

[41] Schneider C, Nobs SP, Kurrer M, Rehrauer H, Thiele C, Kopf M. Induction of the nuclear receptor PPAR-γ by the cytokine GM-CSF is critical for the differentiation of fetal monocytes into alveolar macrophages. Nat Immunol 2014 1511. 2014;15:1026–1037. doi: 10.1038/ni.3005 25263125

[42] Baker AD, Malur A, Barna BP, Ghosh S, Kavuru MS, Malur AG, et al. Targeted PPARγ deficiency in alveolar macrophages disrupts surfactant catabolism. J Lipid Res. 2010;51:1325–31. doi: 10.1194/jlr.M001651 20064973 PMC3035495

[43] Ibrahim-Granet O, Philippe B, Boleti H, Boisvieux-Ulrich E, Grenet D, Stern M, et al. Phagocytosis and intracellular fate of Aspergillus fumigatus conidia in alveolar macrophages. Infect Immun. 2003;71:891–903. doi: 10.1128/IAI.71.2.891-903.2003 12540571 PMC145364

[44] Schaffner A, Douglas H, Braude A. Selective Protection against Conidia by Mononuclear and against Mycelia by Polymorphonuclear Phagocytes in Resistance to Aspergillus: OBSERVATIONS ON THESE TWO LINES OF DEFENSE IN VIVO AND IN VITRO WITH HUMAN AND MOUSE PHAGOCYTES. J Clin Invest. 1982;69:617–31. doi: 10.1172/jci110489 7037853 PMC371019

[45] Wah Wong SS, Rani M, Dodagatta-Marri E, Ibrahim-Granet O, Kishore U, Bayry J, et al. Fungal melanin stimulates surfactant protein D-mediated opsonization of and host immune response to Aspergillus fumigatus spores. J Biol Chem. 2018;293:4901–12. doi: 10.1074/jbc.M117.815852 29414772 PMC5880149

[46] Sun H, Xu XY, Shao HT, Su X, Wu XD, Wang Q, et al. Dectin-2 is predominately macrophage restricted and exhibits conspicuous expression during Aspergillus fumigatus invasion in human lung. Cell Immunol. 2013;284:60–7. doi: 10.1016/j.cellimm.2013.06.013 23928558

[47] Steele C, Rapaka RR, Metz A, Pop SM, Williams DL, Gordon S, et al. The Beta-Glucan Receptor Dectin-1 Recognizes Specific Morphologies of Aspergillus fumigatus. PLoS Pathog. 2005;1:e42. doi: 10.1371/journal.ppat.0010042 16344862 PMC1311140

[48] Herre J, Gordon S, Brown GD. Dectin-1 and its role in the recognition of β-glucans by macrophages. Mol Immunol. 2004;40:869–76. doi: 10.1016/j.molimm.2003.10.007 14698225

[49] Philippe B, Ibrahim-Granet O, Prévost MC, Gougerot-Pocidalo MA, Perez MS, Van der Meeren A, et al. Killing of Aspergillus fumigatus by alveolar macrophages is mediated by reactive oxidant intermediates. Infect Immun. 2003;71:3034–42. doi: 10.1128/IAI.71.6.3034-3042.2003 12761080 PMC155721

[50] Briard B, Fontaine T, Samir P, Place DE, Muszkieta L, Malireddi RKS, et al. Galactosaminogalactan activates the inflammasome to provide host protection. Nat 2020 5887839. 2020;588:688–692. doi: 10.1038/s41586-020-2996-z 33268895 PMC8086055

[51] Leal SM, Cowden S, Hsia YC, Ghannoum MA, Momany M, Pearlman E. Distinct Roles for Dectin-1 and TLR4 in the Pathogenesis of Aspergillus fumigatus Keratitis. PLoS Pathog. 2010;6:e1000976. doi: 10.1371/journal.ppat.1000976 20617171 PMC2895653

[52] Caffrey AK, Lehmann MM, Zickovich JM, Espinosa V, Shepardson KM, Watschke CP, et al. IL-1α Signaling Is Critical for Leukocyte Recruitment after Pulmonary Aspergillus fumigatus Challenge. PLoS Pathog. 2015;11:e1004625. doi: 10.1371/journal.ppat.1004625 25629406 PMC4309569

[53] Meier A, Kirschning CJ, Nikolaus T, Wagner H, Heesemann J, Ebel F. Toll-like receptor (TLR) 2 and TLR4 are essential for Aspergillus-induced activation of murine macrophages. Cell Microbiol. 2003;5:561–70. doi: 10.1046/j.1462-5822.2003.00301.x 12864815

[54] Deerhake ME, Wheaton JD, Parker ME, Juvvadi PR, et al. Functional heterogeneity of alveolar macrophage population based on expression of CXCL2. Sci Immunol. 2020;5. doi: 10.1126/sciimmunol.aba7350 32769172 PMC7717592

[55] Mathie SA, Dixon KL, Walker SA, Tyrrell V, Mondhe M, O’Donnell VB, et al. Alveolar macrophages are sentinels of murine pulmonary homeostasis following inhaled antigen challenge. Allergy. 2015;70:80–9. doi: 10.1111/all.12536 25331546 PMC4283732

[56] Naessens T, Vander Beken S, Bogaert P, Van Rooijen N, Lienenklaus S, Weiss S, et al. Innate imprinting of murine resident alveolar macrophages by allergic bronchial inflammation causes a switch from hypoinflammatory to hyperinflammatory reactivity. Am J Pathol. 2012;181:174–84. doi: 10.1016/j.ajpath.2012.03.015 22613023

[57] Thomassen MJ, Divis LT, Fisher CJ. Regulation of Human Alveolar Macrophage Inflammatory Cytokine Production by Interleukin-10. Clin Immunol Immunopathol. 1996;80:321–4. doi: 10.1006/clin.1996.0130 8811054

[58] Lambrecht BN. Alveolar macrophage in the driver’s seat. Immunity. 2006;24:366–8. doi: 10.1016/j.immuni.2006.03.008 16618595

[59] Coleman MM, Ruane D, Moran B, Dunne PJ, Keane J, Mills KHG. Alveolar macrophages contribute to respiratory tolerance by inducing FoxP3 expression in naive T cells. Am J Respir Cell Mol Biol. 2013;48:773–80. doi: 10.1165/rcmb.2012-0263OC 23492186

[60] Zasłona Z, Przybranowski S, Wilke C, van Rooijen N, Teitz-Tennenbaum S, Osterholzer JJ, et al. Resident Alveolar Macrophages Suppress, whereas Recruited Monocytes Promote, Allergic Lung Inflammation in Murine Models of Asthma. J Immunol. 2014;193:4245–53. doi: 10.4049/jimmunol.1400580 25225663 PMC4185233

[61] Careau E, Turmel V, Lauzon-Joset JF, Bissonnette EY. Alveolar macrophages reduce airway hyperresponsiveness and modulate cytokine levels 2010;36: 255–261. doi: 10.3109/01902140903410757 20497019

[62] Bang BR, Chun E, Shim EJ, Lee HS, Lee SY, Cho SH, et al. Alveolar macrophages modulate allergic inflammation in a murine model of asthma. Exp Mol Med. 2011;43:275–80. doi: 10.3858/emm.2011.43.5.028 21415590 PMC3104249

[63] Song C, Luo L, Lei Z, Li B, Liang Z, Liu G, et al. IL-17-Producing Alveolar Macrophages Mediate Allergic Lung Inflammation Related to Asthma. J Immunol. 2008;181:6117–24. doi: 10.4049/jimmunol.181.9.6117 18941201

[64] Lilly LM, Gessner MA, Dunaway CW, Metz AE, Schwiebert L, Weaver CT, et al. The β-Glucan Receptor Dectin-1 Promotes Lung Immunopathology during Fungal Allergy via IL-22. J Immunol. 2012;189:3653–60. doi: 10.4049/jimmunol.1201797 22933634 PMC3448838

[65] Millien VO, Lu W, Shaw J, Yuan X, Mak G, Roberts L, et al. Cleavage of fibrinogen by proteinases elicits allergic responses through Toll-like receptor 4. Science. 2013;341:792–6. doi: 10.1126/science.1240342 23950537 PMC3898200

[66] Landers CT, Tung HY, Morgan Knight J, Madison MC, Wu Y, Zeng Z, et al. Selective cleavage of fibrinogen by diverse proteinases initiates innate allergic and antifungal immunity through CD11b. J Biol Chem. 2019;294:8834–47. doi: 10.1074/jbc.RA118.006724 30992366 PMC6552423

[67] Fu Z, Akula S, Thorpe M, Hellman L. Highly Selective Cleavage of TH2-Promoting Cytokines by the Human and the Mouse Mast Cell Tryptases, Indicating a Potent Negative Feedback Loop on TH2 Immunity. Int J Mol Sci 2019, Vol 20, Page 5147. 2019;20:5147. doi: 10.3390/ijms20205147 31627390 PMC6834136

[68] Cho M, Lee JE, Lim H, Shin HW, Khalmuratova R, Choi G, et al. Fibrinogen cleavage products and Toll-like receptor 4 promote the generation of programmed cell death 1 ligand 2–positive dendritic cells in allergic asthma. J Allergy Clin Immunol. 2018;142:530–541.e6. doi: 10.1016/j.jaci.2017.09.019 29038008

[69] Schyns J, Bureau F, Marichal T. Lung interstitial macrophages: Past, present, and future. Journal of Immunology Research Hindawi Limited. 2018. doi: 10.1155/2018/5160794 29854841 PMC5952507

[70] Pisu D, Huang L, Narang V, Theriault M, Lê-Bury G, Lee B, et al. Single cell analysis of M. tuberculosis phenotype and macrophage lineages in the infected lung. J Exp Med. 2021;218. doi: 10.1084/jem.20210615 34292313 PMC8302446

[71] Roquilly A, Jacqueline C, Davieau M, Mollé A, Sadek A, Fourgeux C, et al. Alveolar macrophages are epigenetically altered after inflammation, leading to long-term lung immunoparalysis. Nat Immunol 2020 216. 2020;21:636–648. doi: 10.1038/s41590-020-0673-x 32424365

[72] Machiels B, Dourcy M, Xiao X, Javaux J, Mesnil C, Sabatel C, et al. A gammaherpesvirus provides protection against allergic asthma by inducing the replacement of resident alveolar macrophages with regulatory monocytes. Nat Immunol 2017 1812. 2017;18:1310–1320. doi: 10.1038/ni.3857 29035391

[73] Lee YG, Jeong JJ, Nyenhuis S, Berdyshev E, Chung S, Ranjan R, et al. Recruited alveolar macrophages, in response to airway epithelial-derived monocyte chemoattractant protein 1/CCl2, regulate airway inflammation and remodeling in allergic asthma. Am J Respir Cell Mol Biol. 2015;52:772–84. doi: 10.1165/rcmb.2014-0255OC 25360868 PMC4491131

[74] Bedoret D, Wallemacq H, Marichal T, Desmet C, Calvo FQ, Henry E, et al. Lung interstitial macrophages alter dendritic cell functions to prevent airway allergy in mice. J Clin Invest. 2009;119:3723. doi: 10.1172/JCI39717 19907079 PMC2786798

[75] Espinosa V, Jhingran A, Dutta O, Kasahara S, Donnelly R, Du P, et al. Inflammatory Monocytes Orchestrate Innate Antifungal Immunity in the Lung. PLoS Pathog. 2014;10:e1003940. doi: 10.1371/journal.ppat.1003940 24586155 PMC3930594

[76] Hohl TM, Rivera A, Lipuma L, Gallegos A, Shi C, Mack M, et al. Inflammatory monocytes facilitate adaptive CD4 T cell responses during respiratory fungal infection. Cell Host Microbe. 2009;6:470–81. doi: 10.1016/j.chom.2009.10.007 19917501 PMC2785497

[77] Vroman H, Hendriks RW, Kool M. Dendritic Cell Subsets in Asthma: Impaired Tolerance or Exaggerated Inflammation? Front Immunol. 2017;8. doi: 10.3389/fimmu.2017.00941 28848549 PMC5552666

[78] Iwasaki A, Medzhitov R. Control of adaptive immunity by the innate immune system. Nat Immunol 2015 164. 2015;16:343–353. doi: 10.1038/ni.3123 25789684 PMC4507498

[79] Hintzen G, Ohl L, del Rio M-L, Rodriguez-Barbosa J-I, Pabst O, Kocks JR, et al. Induction of Tolerance to Innocuous Inhaled Antigen Relies on a CCR7-Dependent Dendritic Cell-Mediated Antigen Transport to the Bronchial Lymph Node. J Immunol 2006;177: 7346–7354. doi: 10.4049/jimmunol.177.10.7346 17082654

[80] Huang H, Dawicki W, Zhang X, Town J, Gordon JR. Tolerogenic Dendritic Cells Induce CD4+CD25hiFoxp3+ Regulatory T Cell Differentiation from CD4+CD25−/loFoxp3− Effector T Cells. J Immunol. 2010;185:5003–10. doi: 10.4049/jimmunol.0903446 20870943

[81] Bozza S, Gaziano R, Spreca A, Bacci A, Montagnoli C, di Francesco P, et al. Dendritic Cells Transport Conidia and Hyphae of Aspergillus fumigatus from the Airways to the Draining Lymph Nodes and Initiate Disparate Th Responses to the Fungus. J Immunol 2002;168: 1362–1371. doi: 10.4049/jimmunol.168.3.1362 11801677

[82] Percier P, de Prins S, Tima G, Beyaert R, Grooten J, Romano M, et al. Aspergillus fumigatus Recognition by Dendritic Cells Negatively Regulates Allergic Lung Inflammation through a TLR2/MyD88 Pathway. Am J Respir Cell Mol Biol. 2021;64:39–49. doi: 10.1165/rcmb.2020-0083OC 32970964

[83] Cabeza-Cabrerizo M, Cardoso A, Minutti CM, Pereira Da Costa M, Reis E, Sousa C. Dendritic Cells Revisited. Annual Review of Immunology Annual Reviews. 2021:131–66. doi: 10.1146/annurev-immunol-061020-053707 33481643

[84] Tamura T, Tailor P, Yamaoka K, Kong HJ, Tsujimura H, O’Shea JJ, et al. IFN Regulatory Factor-4 and -8 Govern Dendritic Cell Subset Development and Their Functional Diversity. J Immunol. 2005;174:2573–81. doi: 10.4049/jimmunol.174.5.2573 15728463

[85] Williams JW, Tjota MY, Clay BS, Vander Lugt B, Bandukwala HS, Hrusch CL, et al. Transcription factor IRF4 drives dendritic cells to promote Th2 differentiation. Nat Commun 2013 41. 2013;4:1–12. doi: 10.1038/ncomms3990 24356538 PMC4003872

[86] Ho AWS, Prabhu N, Betts RJ, Ge MQ, Dai X, Hutchinson PE, et al. Lung CD103+ Dendritic Cells Efficiently Transport Influenza Virus to the Lymph Node and Load Viral Antigen onto MHC Class I for Presentation to CD8 T Cells. J Immunol. 2011;187:6011–21. doi: 10.4049/jimmunol.1100987 22043017

[87] Mashayekhi M, Sandau MM, Dunay IR, Frickel EM, Khan A, Goldszmid RS, et al. CD8α+ Dendritic Cells Are the Critical Source of Interleukin-12 that Controls Acute Infection by Toxoplasma gondii Tachyzoites. Immunity. 2011;35:249–59. doi: 10.1016/j.immuni.2011.08.008 21867928 PMC3171793

[88] Furuhashi K, Suda T, Hasegawa H, Suzuki Y, Hashimoto D, Enomoto N, et al. Mouse lung CD103 +and CD11b high dendritic cells preferentially induce distinct CD4 + T-cell responses. Am J Respir Cell Mol Biol. 2012;46:165–72. doi: 10.1165/rcmb.2011-0070OC 21908266

[89] Plantinga M, Guilliams M, Vanheerswynghels M, Deswarte K, Branco-Madeira F, Toussaint W, et al. Conventional and Monocyte-Derived CD11b+ Dendritic Cells Initiate and Maintain T Helper 2 Cell-Mediated Immunity to House Dust Mite Allergen. Immunity. 2013;38:322–35. Available: http://www.cell.com/article/S1074761313000046/fulltext. doi: 10.1016/j.immuni.2012.10.016 23352232

[90] Schlitzer A, McGovern N, Teo P, Zelante T, Atarashi K, Low D, et al. IRF4 transcription factor-dependent CD11b+ dendritic cells in human and mouse control mucosal IL-17 cytokine responses. Immunity. 2013;38:970–83. doi: 10.1016/j.immuni.2013.04.011 23706669 PMC3666057

[91] Bosteels C, Neyt K, Vanheerswynghels M, van Helden MJ, Sichien D, Debeuf N, et al. Inflammatory Type 2 cDCs Acquire Features of cDC1s and Macrophages to Orchestrate Immunity to Respiratory Virus Infection. Immunity. 2020;52:1039–1056.e9. doi: 10.1016/j.immuni.2020.04.005 32392463 PMC7207120

[92] Ginhoux F, Guilliams M, Merad M. Expanding dendritic cell nomenclature in the single-cell era. Nat Rev Immunol 2022 222. 2022;22:67–68. doi: 10.1038/s41577-022-00675-7 35027741

[93] León B, Ardavín C. Monocyte-derived dendritic cells in innate and adaptive immunity. Immunol Cell Biol. 2008;86:320–4. doi: 10.1038/icb.2008.14 18362945

[94] Ramirez-Ortiz ZG, Lee CK, Wang JP, Boon L, Specht CA, Levitz SM. A non-redundant role for plasmacytoid dendritic cells in host defense against the human fungal pathogen Aspergillus fumigatus. Cell Host Microbe. 2011;9:415. doi: 10.1016/j.chom.2011.04.007 21575912 PMC3100664

[95] Ramirez-Ortiz ZG, Specht CA, Wang JP, Lee CK, Bartholomeu DC, Gazzinelli RT, et al. Toll-like receptor 9-dependent immune activation by unmethylated CpG motifs in Aspergillus fumigatus DNA. Infect Immun. 2008;76:2123–9. doi: 10.1128/IAI.00047-08 18332208 PMC2346696

[96] Perruccio K, Bozza S, Montagnoli C, Bellocchio S, Aversa F, Martelli M, et al. Prospects for dendritic cell vaccination against fungal infections in hematopoietic transplantation. Blood Cells Mol Dis. 2004;33:248–55. doi: 10.1016/j.bcmd.2004.08.011 15528139

[97] Loures FV, Röhm M, Lee CK, Santos E, Wang JP, Specht CA, et al. Recognition of Aspergillus fumigatus Hyphae by Human Plasmacytoid Dendritic Cells Is Mediated by Dectin-2 and Results in Formation of Extracellular Traps. PLoS Pathog. 2015;11. doi: 10.1371/journal.ppat.1004643 25659141 PMC4450068

[98] Park SY, Jing X, Gupta D, Dziarski R. Peptidoglycan recognition protein 1 enhances experimental asthma by promoting Th2 and Th17 and limiting regulatory T cell and plasmacytoid dendritic cell responses. J Immunol. 2013;190:3480–92. doi: 10.4049/jimmunol.1202675 23420883 PMC3608703

[99] Kool M, van Nimwegen M, Willart MAM, Muskens F, Boon L, Smit JJ, et al. An Anti-Inflammatory Role for Plasmacytoid Dendritic Cells in Allergic Airway Inflammation. J Immunol 2009;183: 1074–1082. doi: 10.4049/jimmunol.0900471 19553531

[100] De Heer HJ, Hammad H, Soullié T, Hijdra D, Vos N, Willart MAM, et al. Essential Role of Lung Plasmacytoid Dendritic Cells in Preventing Asthmatic Reactions to Harmless Inhaled Antigen. J Exp Med. 2004;200:89–98. doi: 10.1084/jem.20040035 15238608 PMC2213319

[101] Matsuse H, Yamagishi T, Kodaka N, Nakano C, Fukushima C, Obase Y, et al. Therapeutic modality of plasmacytoid dendritic cells in a murine model of *Aspergillus fumigatus* sensitized and infected asthma. AIMS Allergy Immunol 2017 4232. 2017;1:232–241. doi: 10.3934/ALLERGY.2017.4.232

[102] Lombardi V, Speak AO, Kerzerho J, Szely N, Akbari O. CD8α^+^β^−^ and CD8α^+^β^+^ plasmacytoid dendritic cells induce Foxp3^+^ regulatory T cells and prevent the induction of airway hyper-reactivity. Mucosal Immunol. 2012;5:432–43. doi: 10.1038/mi.2012.20 22472775 PMC3378819

[103] Chairakaki AD, Saridaki MI, Pyrillou K, Mouratis MA, Koltsida O, Walton RP, et al. Plasmacytoid dendritic cells drive acute asthma exacerbations. J Allergy Clin Immunol. 2018;142:542–556.e12. doi: 10.1016/j.jaci.2017.08.032 29054692

[104] Roy RM, Paes HC, Nanjappa SG, Sorkness R, Gasper D, Sterkel A, et al. Complement component 3C3 and C3a receptor are required in chitin-dependent allergic sensitization to Aspergillus fumigatus but dispensable in chitin-induced innate allergic inflammation. MBio. 2013;4. doi: 10.1128/mBio.00162-13 23549917 PMC3622928

[105] Desch AN, Randolph GJ, Murphy K, Gautier EL, Kedl RM, Lahoud MH, et al. CD103+ pulmonary dendritic cells preferentially acquire and present apoptotic cell–associated antigen. J Exp Med. 2011;208:1789–97. doi: 10.1084/jem.20110538 21859845 PMC3171085

[106] Canton J, Blees H, Henry CM, Buck MD, Schulz O, Rogers NC, et al. The receptor DNGR-1 signals for phagosomal rupture to promote cross-presentation of dead-cell-associated antigens. Nat Immunol 2020 222. 2020;22:140–153. doi: 10.1038/s41590-020-00824-x 33349708 PMC7116638

[107] Conejero L, Khouili SC, Martínez-Cano S, Izquierdo HM, Brandi P, Sancho D. Lung CD103+ dendritic cells restrain allergic airway inflammation through IL-12 production. JCI insight. 2017;2. doi: 10.1172/jci.insight.90420 28515363 PMC5436540

[108] Bernatchez E, Gold MJ, Langlois A, Lemay AM, Brassard J, Flamand N, et al. Pulmonary CD103 expression regulates airway inflammation in asthma. Am J Physiol Lung Cell Mol Physiol. 2015;308:L816–26. doi: 10.1152/ajplung.00319.2014 25681437

[109] Zelante T, Wong AYW, Ping TJ, Chen J, Sumatoh HR, Viganò E, et al. CD103+ Dendritic Cells Control Th17 Cell Function in the Lung. Cell Rep. 2015;12:1789–801. doi: 10.1016/j.celrep.2015.08.030 26365185

[110] Kastelberg B, Tubau-Juni N, Ayubi T, Leung A, Leber A, Hontecillas R, et al. NLRX1 is a key regulator of immune signaling during invasive pulmonary aspergillosis. PLoS Pathog. 2020;16:e1008854. doi: 10.1371/journal.ppat.1008854 32956405 PMC7529209

[111] Break TJ, Hoffman KW, Swamydas M, Lee CCR, Lim JK, Lionakis MS. Batf3-dependent CD103+ dendritic cell accumulation is dispensable for mucosal and systemic antifungal host defense. Virulence. 2016;7:826. doi: 10.1080/21505594.2016.1186324 27191829 PMC5029292

[112] Van Prooyen N, Henderson CA, Hocking Murray D, Sil A. CD103+ Conventional Dendritic Cells Are Critical for TLR7/9-Dependent Host Defense against Histoplasma capsulatum, an Endemic Fungal Pathogen of Humans. PLoS Pathog. 2016;12. doi: 10.1371/journal.ppat.1005749 27459510 PMC4961300

[113] Norimoto A, Hirose K, Iwata A, Tamachi T, Yokota M, Takahashi K, et al. Dectin-2 promotes house dust mite-induced T helper type 2 and type 17 cell differentiation and allergic airway inflammation in mice. Am J Respir Cell Mol Biol. 2014;51:201–9. doi: 10.1165/rcmb.2013-0522OC 24588637

[114] Lee J, Zhang J, Chung YJ, Kim JH, Kook CM, González-Navajas JM, et al. Inhibition of IRF4 in dendritic cells by PRR-independent and-dependent signals inhibit Th2 and promote Th17 responses. Elife. 2020;9. doi: 10.7554/eLife.49416 32014112 PMC7000221

[115] Sakurai S, Furuhashi K, Horiguchi R, Nihashi F, Yasui H, Karayama M, et al. Conventional type 2 lung dendritic cells are potent inducers of follicular helper T cells in the asthmatic lung. Allergol Int. 2021;70:351–9. doi: 10.1016/j.alit.2021.01.008 33674189

[116] Gao Y, Nish SA, Jiang R, Hou L, Licona-Limón P, Weinstein JS, et al. Control of T Helper 2 Responses by Transcription Factor IRF4-Dependent Dendritic Cells. Immunity. 2013;39:722–32. doi: 10.1016/j.immuni.2013.08.028 24076050 PMC4110745

[117] Van Rijt LS, Prins JB, Leenen PJM, Thielemans K, De Vries VC, Hoogsteden HC, et al. Allergen-induced accumulation of airway dendritic cells is supported by an increase in CD31hiLy-6Cneg bone marrow precursors in a mouse model of asthma. Blood. 2002;100:3663–71. doi: 10.1182/blood-2002-03-0673 12393720

[118] Wiesner DL, Specht CA, Lee CK, Smith KD, Mukaremera L, Lee ST, et al. Chitin Recognition via Chitotriosidase Promotes Pathologic Type-2 Helper T Cell Responses to Cryptococcal Infection. PLoS Pathog. 2015;11:e1004701. doi: 10.1371/journal.ppat.1004701 25764512 PMC4357429

[119] Medoff BD, Seung E, Hong S, Thomas SY, Sandall BP, Duffield JS, et al. CD11b+ Myeloid Cells Are the Key Mediators of Th2 Cell Homing into the Airway in Allergic Inflammation. J Immunol. 2009;182:623–35. doi: 10.4049/jimmunol.182.1.623 19109196 PMC2718444

[120] Perros F, Hoogsteden HC, Coyle AJ, Lambrecht BN, Hammad H. Blockade of CCR4 in a humanized model of asthma reveals a critical role for DC-derived CCL17 and CCL22 in attracting Th2 cells and inducing airway inflammation. Allergy. 2009;64:995–1002. doi: 10.1111/j.1398-9995.2009.02095.x 19630858

[121] Mansouri S, Katikaneni DS, Gogoi H, Pipkin M, Machuca TN, Emtiazjoo AM, et al. Lung IFNAR1hi TNFR2+ cDC2 promotes lung regulatory T cells induction and maintains lung mucosal tolerance at steady state. Mucosal Immunol. 2020;13:595. doi: 10.1038/s41385-020-0254-1 31959883 PMC7311323

[122] Mansouri S, Gogoi H, Pipkin M, Machuca TN, Emtiazjoo AM, Sharma AK, et al. In vivo reprogramming of pathogenic lung TNFR2+ cDC2s by IFNβ inhibits HDM-induced asthma. Sci Immunol. 2021;6:8472. doi: 10.1126/sciimmunol.abi8472 34244314 PMC8323989

[123] Raymond M, Rubio M, Fortin G, Shalaby KH, Hammad H, Lambrecht BN, et al. Selective control of SIRP-α–positive airway dendritic cell trafficking through CD47 is critical for the development of TH2-mediated allergic inflammation. J Allergy Clin Immunol. 2009;124:1333–1342.e1. doi: 10.1016/j.jaci.2009.07.021 19748659

[124] Guo Y, Kasahara S, Jhingran A, Tosini NL, Zhai B, Aufiero MA, et al. During Aspergillus Infection, Monocyte-Derived DCs, Neutrophils, and Plasmacytoid DCs Enhance Innate Immune Defense through CXCR3-Dependent Crosstalk. Cell Host Microbe. 2020;28:104–116.e4. doi: 10.1016/j.chom.2020.05.002 32485165 PMC7263227

[125] Fei M, Bhatia S, Oriss TB, Yarlagadda M, Khare A, Akira S, et al. TNF-α from inflammatory dendritic cells (DCs) regulates lung IL-17A/IL-5 levels and neutrophilia versus eosinophilia during persistent fungal infection. Proc Natl Acad Sci U S A. 2011;108:5360–5. doi: 10.1073/pnas.1015476108 21402950 PMC3069210

[126] Dutertre CA, Becht E, Irac SE, Khalilnezhad A, Narang V, Khalilnezhad S, et al. Single-Cell Analysis of Human Mononuclear Phagocytes Reveals Subset-Defining Markers and Identifies Circulating Inflammatory Dendritic Cells. Immunity. 2019;51:573–589.e8. doi: 10.1016/j.immuni.2019.08.008 31474513

[127] Cytlak U, Resteu A, Pagan S, Green K, Milne P, Maisuria S, et al. Differential IRF8 Transcription Factor Requirement Defines Two Pathways of Dendritic Cell Development in Humans. Immunity. 2020;53:353–370.e8. doi: 10.1016/j.immuni.2020.07.003 32735845 PMC7447982

[128] Brown CC, Gudjonson H, Pritykin Y, Deep D, Lavallée VP, Mendoza A, et al. Transcriptional Basis of Mouse and Human Dendritic Cell Heterogeneity. Cell. 2019;179:846. doi: 10.1016/j.cell.2019.09.035 31668803 PMC6838684

[129] GeurtsvanKessel CH, Lambrecht BN. Division of labor between dendritic cell subsets of the lung. Mucosal Immunology. Nature Publishing Group; 2008. pp. 442–450. doi: 10.1038/mi.2008.39 19079211

[130] Bosteels C, Fierens K, De Prijck S, Van Moorleghem J, Vanheerswynghels M, De Wolf C, et al. CCR2- and Flt3-Dependent Inflammatory Conventional Type 2 Dendritic Cells Are Necessary for the Induction of Adaptive Immunity by the Human Vaccine Adjuvant System AS01. Front Immunol. 2021;11. doi: 10.3389/fimmu.2020.606805 33519816 PMC7841299

[131] Cohen M, Giladi A, Gorki AD, Solodkin DG, Zada M, Hladik A, et al. Lung Single-Cell Signaling Interaction Map Reveals Basophil Role in Macrophage Imprinting. Cell. 2018;175:1031–1044.e18. doi: 10.1016/j.cell.2018.09.009 30318149

[132] McCowan J, Fercoq F, Kirkwood PM, T’Jonck W, Hegarty LM, Mawer CM, et al. The transcription factor EGR2 is indispensable for tissue-specific imprinting of alveolar macrophages in health and tissue repair. Sci Immunol. 2021;6:2132. doi: 10.1126/sciimmunol.abj2132 34797692 PMC7612216

[133] Papaioannou NE, Salei N, Rambichler S, Ravi K, Popovic J, Küntzel V, et al. Environmental signals rather than layered ontogeny imprint the function of type 2 conventional dendritic cells in young and adult mice. Nat Commun 2021 121. 2021;12:1–20. doi: 10.1038/s41467-020-20659-2 33469015 PMC7815729

[134] Van Dyken SJ, Locksley RM. Interleukin-4-and interleukin-13-mediated alternatively activated macrophages: Roles in homeostasis and disease. Annual Review of Immunology Annual Reviews. 2013:317–43. doi: 10.1146/annurev-immunol-032712-095906 23298208 PMC3606684

[135] Sutherland TE, Rückerl D, Logan N, Duncan S, Wynn TA, Allen JE. Ym1 induces RELMα and rescues IL-4Rα deficiency in lung repair during nematode infection. PLoS Pathog. 2018;14:e1007423. doi: 10.1371/journal.ppat.1007423 30500858 PMC6291165

[136] Loke P, Nair MG, Parkinson J, Guiliano D, Blaxter M, Allen JE. IL-4 dependent alternatively-activated macrophages have a distinctive in vivo gene expression phenotype. BMC Immunol. 2002;3:1–11. doi: 10.1186/1471-2172-3-7/FIGURES/3 12098359 PMC117781

[137] Welch JS, Escoubet-Lozach L, Sykes DB, Liddiard K, Greaves DR, Glass CK. TH2 Cytokines and Allergic Challenge Induce Ym1 Expression in Macrophages by a STAT6-dependent Mechanism. J Biol Chem. 2002;277:42821–9. doi: 10.1074/jbc.M205873200 12215441

[138] Faas M, Ipseiz N, Ackermann J, Culemann S, Grüneboom A, Schröder F, et al. IL-33-induced metabolic reprogramming controls the differentiation of alternatively activated macrophages and the resolution of inflammation. Immunity. 2021;54:2531–2546.e5. doi: 10.1016/j.immuni.2021.09.010 34644537 PMC7617137

[139] Bosurgi L, Cao YG, Cabeza-Cabrerizo M, Tucci A, Hughes LD, Kong Y, et al. Macrophage function in tissue repair and remodeling requires IL-4 or IL-13 with apoptotic cells. Science (80-). 2017;356:1072–6. doi: 10.1126/science.aai8132 28495875 PMC5556699

[140] Minutti CM, Jackson-Jones LH, García-Fojeda B, Knipper JA, Sutherland TE, Logan N, et al. Local amplifiers of IL-4Ra-mediated macrophage activation promote repair in lung and liver. Science (80-). 2017;356:1076–80. doi: 10.1126/SCIENCE.AAJ2067/SUPPL_FILE/AAJ2067_MINUTTI_SM.PDF PMC5737834 28495878

[141] Wagener J, Maccallum DM, Brown GD, Gow NAR. Candida albicans chitin increases arginase-1 activity in human macrophages, with an impact on macrophage antimicrobial functions. MBio. 2017;8. doi: 10.1128/mBio.01820-16 28119468 PMC5263244

[142] Bhatia S, Fei M, Yarlagadda M, Qi Z, Akira S, Saijo S, et al. Rapid Host Defense against Aspergillus fumigatus Involves Alveolar Macrophages with a Predominance of Alternatively Activated Phenotype. PLoS ONE. 2011;6. doi: 10.1371/journal.pone.0015943 21246055 PMC3016416

[143] Moreira AP, Cavassani KA, Hullinger R, Rosada RS, Fong DJ, Murray L, et al. Serum amyloid P attenuates M2 macrophage activation and protects against fungal spore–induced allergic airway disease. J Allergy Clin Immunol. 2010;126:712–721.e7. doi: 10.1016/j.jaci.2010.06.010 20673988

[144] Svedberg FR, Brown SL, Krauss MZ, Campbell L, Sharpe C, Clausen M, et al. The lung environment controls alveolar macrophage metabolism and responsiveness in type 2 inflammation. Nat Immunol. 2019;20:571. doi: 10.1038/s41590-019-0352-y 30936493 PMC8381729

[145] Halim TYF, Hwang YY, Scanlon ST, Zaghouani H, Garbi N, Fallon PG, et al. Group 2 innate lymphoid cells license dendritic cells to potentiate memory TH2 cell responses. Nat Immunol. 2016;17:57–64. doi: 10.1038/ni.3294 26523868 PMC4685755

[146] Besnard AG, Togbe D, Guillou N, Erard F, Quesniaux V, Ryffel B. IL-33-activated dendritic cells are critical for allergic airway inflammation. Eur J Immunol. 2011;41:1675–86. doi: 10.1002/eji.201041033 21469105

[147] Mayer JU, Hilligan KL, Chandler JS, Eccles DA, Old SI, Domingues RG, et al. Homeostatic IL-13 in healthy skin directs dendritic cell differentiation to promote TH2 and inhibit TH17 cell polarization. Nat Immunol 2021 2212. 2021;22:1538–1550. doi: 10.1038/s41590-021-01067-0 34795444

[148] DUNNILL MS. The pathology of asthma, with special reference to changes in the bronchial mucosa. J Clin Pathol. 1960;13:27–33. doi: 10.1136/jcp.13.1.27 13818688 PMC479992

[149] Haczku A, Atochina EN, Tomer Y, Chen H, Scanlon ST, Russo S, et al. Aspergillus fumigatus-Induced Allergic Airway Inflammation Alters Surfactant Homeostasis and Lung Function in BALB/c Mice 2012;25: 45–50. doi: 10.1165/AJRCMB.25.1.4391 11472974

[150] Koopmans JG, Van Der Zee JS, Krop EJM, Lopuhaä CE, Jansen HM, Batenburg JJ. Serum surfactant protein D is elevated in allergic patients. Clin Exp Allergy. 2004;34:1827–33. doi: 10.1111/j.1365-2222.2004.02083.x 15663555

[151] Cheng G, Ueda T, Numao T, Kuroki Y, Nakajima H, Fukushima Y, et al. Increased levels of surfactant protein A and D in bronchoalveolar lavage fluids in patients with bronchial asthma. Eur Respir J. 2000;16:831–5. doi: 10.1183/09031936.00.16583100 11153579

[152] Erpenbeck VJ, Schmidt R, Günther A, Krug N, Hohlfeld JM. Surfactant protein levels in bronchoalveolar lavage after segmental allergen challenge in patients with asthma. Allergy. 2006;61:598–604. doi: 10.1111/j.1398-9995.2006.01062.x 16629790

[153] Evans CM, Williams OW, Tuvim MJ, Nigam R, Mixides GP, Blackburn MR, et al. Mucin is produced by clara cells in the proximal airways of antigen-challenged mice. Am J Respir Cell Mol Biol. 2004;31:382–94. doi: 10.1165/rcmb.2004-0060OC 15191915 PMC10862391

[154] Ordoñez CL, Khashayar R, Wong HH, Ferrando R, Wu R, Hyde DM, et al. Mild and moderate asthma is associated with airway goblet cell hyperplasia and abnormalities in mucin gene expression. Am J Respir Crit Care Med. 2001;163:517–23. doi: 10.1164/ajrccm.163.2.2004039 11179133

[155] Kuyper LM, Paré PD, Hogg JC, Lambert RK, Ionescu D, Woods R, et al. Characterization of airway plugging in fatal asthma. Am J Med. 2003;115:6–11. doi: 10.1016/s0002-9343(03)00241-9 12867228

[156] Dunican EM, Elicker BM, Gierada DS, Nagle SK, Schiebler ML, Newell JD, et al. Mucus plugs in patients with asthma linked to eosinophilia and airflow obstruction. J Clin Invest. 2018;128:997–1009. doi: 10.1172/JCI95693 29400693 PMC5824874

[157] Morgan LE, Jaramillo AM, Shenoy SK, Raclawska D, Emezienna NA, Richardson VL, et al. Disulfide disruption reverses mucus dysfunction in allergic airway disease. Nat Commun 2021 121. 2021;12:1–9. doi: 10.1038/s41467-020-20499-0 33431872 PMC7801631

[158] Evans CM, Raclawska DS, Ttofali F, Liptzin DR, Fletcher AA, Harper DN, et al. The polymeric mucin Muc5ac is required for allergic airway hyperreactivity. Nat Commun. 2015;6. doi: 10.1038/ncomms7281 25687754 PMC4333679

[159] Kerr SC, Fischer GJ, Sinha M, McCabe O, Palmer JM, Choera T, et al. FleA Expression in Aspergillus fumigatus Is Recognized by Fucosylated Structures on Mucins and Macrophages to Prevent Lung Infection. PLoS Pathog. 2016;12:e1005555. doi: 10.1371/journal.ppat.1005555 27058347 PMC4825926

[160] Rivera CA, Randrian V, Richer W, Gerber-Ferder Y, Delgado MG, Chikina AS, et al. Epithelial colonization by gut dendritic cells promotes their functional diversification. Immunity. 2022;55:129–144.e8. doi: 10.1016/j.immuni.2021.11.008 34910930 PMC8751639

[161] Melo-Gonzalez F, Fenton TM, Forss C, Smedley C, Goenka A, MacDonald AS, et al. Intestinal mucin activates human dendritic cells and IL-8 production in a glycan-specific manner. J Biol Chem. 2018;293:8543. doi: 10.1074/jbc.M117.789305 29581231 PMC5986209

[162] Persson EK, Verstraete K, Heyndrickx I, Gevaert E, Aegerter H, Percier JM, et al. Protein crystallization promotes type 2 immunity and is reversible by antibody treatment. Science. 2019;364. doi: 10.1126/science.aaw4295 31123109

[163] Mishra A. Metabolic Plasticity in Dendritic Cell Responses: Implications in Allergic Asthma. J Immunol Res. 2017;2017. doi: 10.1155/2017/5134760 29387732 PMC5745769

[164] Ogger PP, Byrne AJ. Macrophage metabolic reprogramming during chronic lung disease. Mucosal Immunol 2020 142. 2020;14:282–295. doi: 10.1038/s41385-020-00356-5 33184475 PMC7658438

[165] Everts B, Amiel E, Huang SCC, Smith AM, Chang CH, Lam WY, et al. TLR-driven early glycolytic reprogramming via the kinases TBK1-IKKε supports the anabolic demands of dendritic cell activation. Nat Immunol. 2014;15:323. doi: 10.1038/ni.2833 24562310 PMC4358322

[166] Viola A, Munari F, Sánchez-Rodríguez R, Scolaro T, Castegna A. The metabolic signature of macrophage responses. Front Immunol. 2019;10:1462. doi: 10.3389/fimmu.2019.01462 31333642 PMC6618143

[167] Srivastava M, Bencurova E, Gupta SK, Weiss E, Löffler J, Dandekar T. Aspergillus fumigatus challenged by human dendritic cells: Metabolic and regulatory pathway responses testify a tight battle. Front Cell Infect Microbiol. 2019;9:168. doi: 10.3389/fcimb.2019.00168 31192161 PMC6540932

[168] Thwe PM, Fritz DI, Snyder JP, Smith PR, Curtis KD, O’Donnell A, et al. Syk-dependent glycolytic reprogramming in dendritic cells regulates IL-1β production to β-glucan ligands in a TLR-independent manner. J Leukoc Biol. 2019;106:1325–35. doi: 10.1002/JLB.3A0819-207RR 31509298 PMC6883127

[169] Gonçalves SM, Duarte-Oliveira C, Campos CF, Aimanianda V, ter Horst R, Leite L, et al. Phagosomal removal of fungal melanin reprograms macrophage metabolism to promote antifungal immunity. Nat Commun 2020 111. 2020;11:1–15. doi: 10.1038/s41467-020-16120-z 32385235 PMC7210971

[170] Baker EH, Baines DL. Airway Glucose Homeostasis: A New Target in the Prevention and Treatment of Pulmonary Infection. Chest. 2018;153:507–14. doi: 10.1016/j.chest.2017.05.031 28610911

[171] Balenga NA, Klichinsky M, Xie Z, Chan EC, Zhao M, Jude J, et al. A fungal protease allergen provokes airway hyperresponsiveness in asthma. Nat Commun. 2015;6:6763. doi: 10.1038/ncomms7763 25865874 PMC4396684

[172] Robinson BWS, Venaille TJ, Mendis AHW, McAleer R. Allergens as proteases: An aspergillus fumigatus proteinase directly induces human epithelial cell detachment. J Allergy Clin Immunol. 1990;86:726–31. doi: 10.1016/s0091-6749(05)80176-9 2229838

[173] Wiesner DL, Merkhofer RM, Ober C, Kujoth GC, Niu M, Keller NP, et al. Club Cell TRPV4 Serves as a Damage Sensor Driving Lung Allergic Inflammation. Cell Host Microbe. 2020;27:614. doi: 10.1016/j.chom.2020.02.006 32130954 PMC7305569

[174] Rowley J, Namvar S, Gago S, Labram B, Bowyer P, Richardson MD, et al. Differential Proinflammatory Responses to Aspergillus fumigatus by Airway Epithelial Cells In Vitro Are Protease Dependent. J fungi (Basel, Switzerland). 2021;7. doi: 10.3390/jof7060468 34200666 PMC8228831

[175] Hiraishi Y, Yamaguchi S, Yoshizaki T, Nambu A, Shimura E, Takamori A, et al. IL-33, IL-25 and TSLP contribute to development of fungal-associated protease-induced innate-type airway inflammation. Sci Rep. 2018;8. doi: 10.1038/s41598-018-36440-x 30575775 PMC6303299

[176] Tomee JFC, Wierenga ATJ, Hiemstra PS, Kauffman HF. Proteases from Aspergillus fumigatus Induce Release of Proinflammatory Cytokines and Cell Detachment in Airway Epithelial Cell Lines. J Infect Dis. 1997;176:300–3. doi: 10.1086/517272 9207388

[177] Kool M, Willart MAM, van Nimwegen M, Bergen I, Pouliot P, Virchow JC, et al. An Unexpected Role for Uric Acid as an Inducer of T Helper 2 Cell Immunity to Inhaled Antigens and Inflammatory Mediator of Allergic Asthma. Immunity. 2011;34:527–40. doi: 10.1016/j.immuni.2011.03.015 21474346

[178] Van Dyken SJ, Nussbaum JC, Lee J, Molofsky AB, Liang HE, Pollack JL, et al. A tissue checkpoint regulates type 2 immunity. Nat Immunol. 2016;17:1381. doi: 10.1038/ni.3582 27749840 PMC5275767

[179] Soumelis V, Reche PA, Kanzler H, Yuan W, Edward G, Homey B, et al. Human epithelial cells trigger dendritic cell–mediated allergic inflammation by producing TSLP. Nat Immunol 2002 37. 2002;3:673–680. doi: 10.1038/ni805 12055625

[180] Halim TYF, Steer CA, Mathä L, Gold MJ, Martinez-Gonzalez I, McNagny KM, et al. Group 2 Innate Lymphoid Cells Are Critical for the Initiation of Adaptive T Helper 2 Cell-Mediated Allergic Lung Inflammation. Immunity. 2014;40:425–35. doi: 10.1016/j.immuni.2014.01.011 24613091 PMC4210641

[181] Ricardo-Gonzalez RR, Van Dyken SJ, Schneider C, Lee J, Nussbaum JC, Liang HE, et al. Tissue signals imprint ILC2 identity with anticipatory function. Nat Immunol 2018 1910. 2018;19:1093–1099. doi: 10.1038/s41590-018-0201-4 30201992 PMC6202223

[182] Wu Y, Zeng Z, Guo Y, Song L, Weatherhead JE, Huang X, et al. Candida albicans elicits protective allergic responses via platelet mediated T helper 2 and T helper 17 cell polarization. Immunity. 2021;54:2595–2610.e7. doi: 10.1016/j.immuni.2021.08.009 34506733 PMC8585696

[183] Shah A, Kannambath S, Herbst S, Rogers A, Soresi S, Carby M, et al. Calcineurin orchestrates lateral transfer of aspergillus fumigatus during macrophage cell death. Am J Respir Crit Care Med. 2016;194:1127–39. doi: 10.1164/rccm.201601-0070OC 27163634 PMC5114448

[184] Khosravi AR, Alheidary S, Nikaein D, Asghari N. Aspergillus fumigatus conidia stimulate lung epithelial cells (TC-1 JHU-1) to produce IL-12, IFNγ, IL-13 and IL-17 cytokines: Modulatory effect of propolis extract. J Mycol Med. 2018;28:594–8. doi: 10.1016/j.mycmed.2018.09.006 30360945

